# Agenesis of Submandibular Glands: A Report of Two Cases with Review of Literature

**DOI:** 10.1155/2014/569026

**Published:** 2014-09-01

**Authors:** Medine Kara, Oğuz Güçlü, Fevzi Sefa Dereköy, Mustafa Resorlu, Gürhan Adam

**Affiliations:** ^1^Department of Otolaryngology, Canakkale Onsekiz Mart University Medical Hospital, Canakkale, Turkey; ^2^Department of Radiology, Canakkale Onsekiz Mart University Medical Hospital, Canakkale, Turkey

## Abstract

*Background*. Congenital absence of the submandibular gland (SMG) is a rare condition. Although complaints such as dry mouth, dental problems, or difficulty in swallowing may be seen, the subjects may also be asymptomatic. The absence of the SMG may be associated with hypertrophy of the contralateral SMG. *Case Report*. We report the case of a 44-year-old woman with incidentally detected left SMG aplasia, with contralateral SMG hypertrophy mimicking a mass, and the case of a 46-year-old woman with incidentally detected bilateral SMG aplasia, demonstrated by computerized tomography (CT) and magnetic resonance imaging (MRI). *Conclusion*. It is important for the clinician to know that this very rare abnormality may exist. When such a case is encountered, symptoms and findings should be reevaluated and, if necessary, conservative therapy should be initiated. The possibility of observing additional deformities should be kept in mind and an evaluation should be done for other cases in the family.

## 1. Introduction

Congenital absence of the submandibular gland (SMG) is a rare condition. The term “aplasia” is described as the total or partial agenesis of the gland [1]. In the English medical literature, approximately 40 cases have been reported to date. The first case was presented in 1885 by Gruber and was a bilateral SMG aplasia [2]. Although its etiology is not known, it is thought to result from a defect that occurs during fetal development. In such subjects, additional developmental deformities may also be observed. Clinical syndromes, such as lacrimo-auriculo-dento-digital syndrome and mandibulofacial-dysostosis (Treacher-Collins syndrome), may also be seen [3]. Unilateral SMG aplasia is often asymptomatic and is usually discovered incidentally through imaging [4].

Due to an inadequate amount of saliva in these cases, some symptoms such as dry mouth, dental problems, and difficulty swallowing are seen. However, while asymptomatic subjects constitute approximately half of the cases, there are also subjects that are arbitrarily diagnosed.

In this study, two cases—a patient with bilateral submandibular aplasia and a patient with unilateral submandibular aplasia—are reported and discussed based on the literature.

## 2. First Case

**Figure 1:**
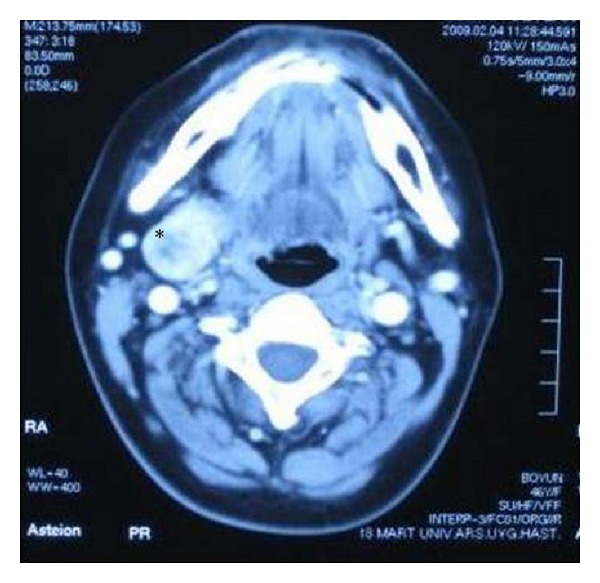
Thyroid mass extension to submandibular area in the first case. “∗” The thyroid mass.

**Figure 2:**
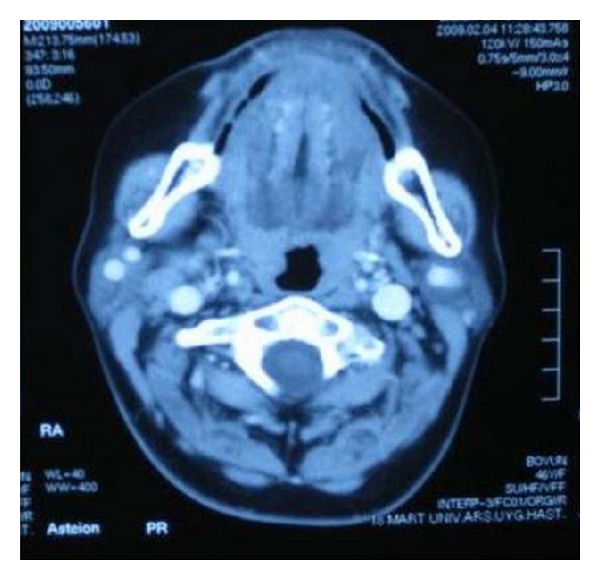
Bilateral absence of submandibular gland in the first case.

A 46-year-old female patient was presented to our clinic with a palpable mass. In the physical examination, her thyroid gland was palpated as bilateral hypertrophic. In the palpation, nodular formations were detected in both lobes. The thyroid hormone levels were within normal limits. In the ultrasound (US) examination, the right lobe of the thyroid was measured at 13 × 4 × 4 cm and the left lobe was measured at 9 × 3 × 3 cm. Additionally, the isoechoic nodular structures were observed, the largest of which was on the right with a diameter of 32 mm while the left had a diameter of 26 × 19 mm with retrosternal extension. In the nodular structures, peripheral and intranodular bleeding was observed. A fine-needle aspiration biopsy was performed and the result showed a benign cytology. To determine its relation with the surrounding tissue, a computerized tomography (CT) of the neck was requested. In the CT scan, it was observed that the thyroid gland extended into the submandibular area and the SMG was bilaterally absent (Figures 1 and 2). Thereafter, the patient was asked if she had a dry mouth, difficulty swallowing, or dental problems, which may occur due to the absence of salivary glands. The oral cavity was reevaluated and the presence of Wharton channel and orifice was examined. A search was done for the presence of an additional deformity, but no additional pathology was detected. The family of the patient did not have any history of this clinical presentation.

## 3. Second Case

**Figure 3:**
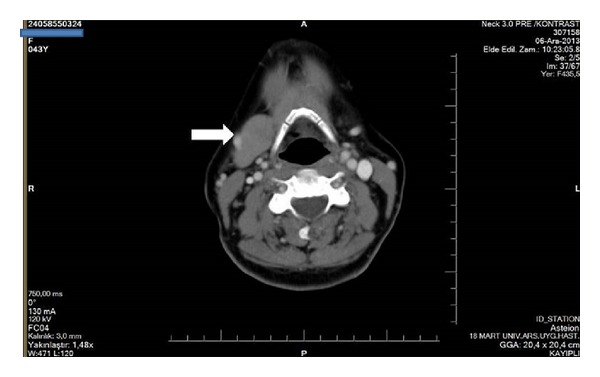
Noncontrast computed tomography axial sections demonstrate absence of left submandibular gland in the second case. White arrow shows the right submandibular gland.

**Figure 4:**
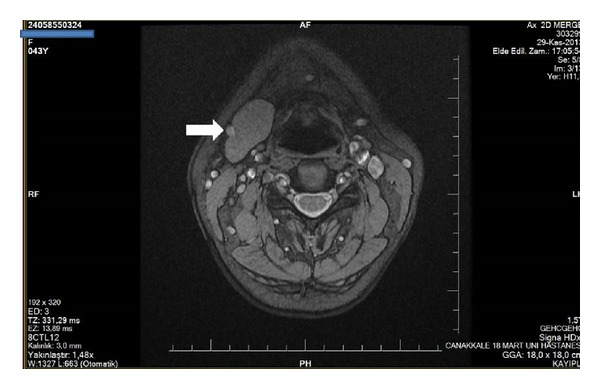
Magnetic resonance image axial sections demonstrate absence of left submandibular gland in the second case. White arrow shows the right submandibular gland.

A 44-year-old female patient complaining of neck pain with a cervical magnetic resonance imaging (MRI) agenesis of the left SMG was consulted in our clinic. On physical examination, no palpable mass was detected in the head and neck region. She had no specific relevant medical history such as dry mouth, dysphagia, teeth and gum problems, or sialadenitis. Agenesis of the left SMG and compensatory hypertrophy in the right SMG were observed in the CT and MRI scans (Figures 3 and 4).

## 4. Discussion

In the literature (Table 1), there is no information about the incidence of the congenital absence of the major SMG; however, to date, approximately 40 cases have been reported [2]. Salivary glands may show unilateral or bilateral aplasia and aplasia of one or more groups. Total or partial agenesis may be observed [3]. Although the etiology of congenital absence of the SMG is unknown, it is thought to result from a defect occurring during fetal development [5]. Submandibular aplasia may also accompany deformities related to the first and second branchial arch abnormalities. Genetic syndromes may also be seen, such as Treacher-Collins syndrome, hemifacial microsomia, ectodermal dysplasia, and lacrimo-auriculo-dento-digital syndrome. In these cases, autosomal dominant inheritance is thought to exist. In the examinations performed in our cases, no additional deformity was detected; however, in cases with more than one salivary gland involved, it is important to screen for other deformities and give genetic counseling if necessary. Furthermore, in cases with developmental deformity, salivary gland abnormalities may also be investigated.

In aplastic cases, dry mouth, difficulty swallowing, and dental problems may be observed. However, approximately half of the cases are asymptomatic [4]. In the literature, there are also some cases that show a compensatory hypertrophy in other glands [5, 6]. In our second case, there was a compensatory hypertrophy in the right SMG. The decreased amount of saliva may also cause impaired oral hygiene and increase the incidence of opportunistic infections. It causes angular cheilitis near the lips, a decreased sense of taste, and gingival problems. In cases with these kinds of symptoms, although the incidence is low, salivary gland aplasia should be considered in a differential diagnosis. Both of our cases were detected incidentally. After determining the existing condition of our patients, they did not describe any additional problems in subsequent questioning. In this regard, we think that the absence of symptoms was caused by an adequate flow of saliva ensured by other glands.

Although, bimanual palpation is absolutely necessary in the diagnosis of SMG aplasia, it does not provide adequate information. Evaluating the presence of the Wharton channel and its orifice is necessary. In total agenesis, the orifice and channel may not be observed. In fact, the diagnosis is determined radiologically. The methods that may be preferred in this area include US, CT, scintigraphy, MRI, and sialography [4]. As some confusion may be occasionally experienced in unilateral aplastic cases or in cases with a compensatory hyperplasia in other glands, having an experienced radiologist is very important. In our first case, a CT was requested for a stage three goiter, which incidentally revealed bilateral SMG aplasia. In the CT scan, it was observed that the thyroid gland extended into the submandibular area. When only these sections were examined, the SMG was clearly observed.

A differential diagnosis of the mass in the submandibular area includes nonmalignant or malignant growths. Nonmalignant swelling may occur as sialadenitis, Sjögren syndrome, cysts, infections, and lymphadenopathy. Neoplastic growths such as the SMG, the tail parotid gland, the Hodgkin's disease, non-Hodgkin's lymphomas, and metastatic disease may be seen [7]. Some rare cases of submandibular swelling have been seen as submandibular localizations of thyroid lesions [8].

In summary, it is important for the clinician to know that this very rare abnormality may exist. When such a case is encountered, symptoms and findings should be reevaluated and, if necessary, conservative therapy should be initiated. The possibility of observing additional deformities should be kept in mind and an evaluation should be done for other cases in the family.

## Figures and Tables

**Table 1:** A literature review of the case reports with absence of the submandibular gland.

Literature	Patient	Presenting symptoms	The way of diagnoses	Findings
Park et al. [1] 2013	A 56-year-old woman	A palpable mass in the left submandibular area	MRI and US	The right SMG was absent and replaced with fatty tissue

Yilmaz et al. [2] 2002	A 32-year-old woman	Pain and tenderness in the left submandibular region and the angle of the mandible	CT, US, and sialography	Absence of the right SMG and a slightly enlarged left SMG

Roh [4] 2006	A 67-year-old man	Hoarseness for 10 months	CT scan of the neck	Absence of the right SMG

Mathison and Hudgins [5] 2008	A 34-year-old female	Stage IIIA nodular sclerosing Hodgkin lymphoma, in the routine control examination	A CT scan of the neck with contrast	Aplasia of bilateral SMGs and moderate enlargement of bilateral SLG

Srinivasan et al. [6] 2006	A 35-year-old woman	Facial pressure and rhinorrhea	CT scan of the paranasal sinuses	Absence of the right SMG, with a “mass” in the right sublingual space in the expected location of the right SLG

Kubo et al. [9] 1990	A 34-year-old male	Pain radiating from his right mandibular body to the temporal region	CT was undertaken to examine his cerebral status	Absence of the right SMG

Fracaro et al. [10] 2002	A 13-year-old female	Severe progressive dental caries and enamel demineralization of the permanent mandibular incisor teeth	Scintigraphy with technetium-99m pertechnetate	Absence of bilateral SMGs

Gallego et al. [11] 2009	A 35-year-old woman	Dry lips and mouth, difficulty with swallowing solid foods, changes in taste, and occasional angular cheilitis over the previous four years	CT	Complete aplasia of the right SMG with compensatory hypertrophy of other mayor salivary glands

Koo et al. [12] 2009	A 48-year-old woman and	Cervical lymphadenopathy.	CT	The left SMG was absent and the area was replaced by fat
	a 42-year-old woman	Palpable mass in the floor of the mouth	CT	The right SMG was absent and the area was replaced by fat

Gupta et al. [13] 2009	A 35-year-old woman and a 7-year-old boy	Prominent posterior cervical lymphadenopathy Symptoms consistent with viral illness	CT and MRI	Both patients were noted to have unilateral aplasia of the right SMG and hypertrophy of the opposite gland

Damar et al. [14] 2013	A 55-year-old female	Myogenic pain radiating from the left shoulder to the left neck and hoarseness	US	Unilateral aplasia of the left SMG

Yerli [15] 2014	A 19-year-old woman	A mass in left submandibular area	US	Aplasia of the left SMG and compensatory hypertrophy of the left SLG

Aiyappan et al. [16] 2010	A 60-year-old woman	Symptoms of sudden-onset left-sided hemiplegia	Nonenhanced CT examination	Absence of the left SMG and few small lymph nodes were seen in the left submandibular triangle

Haktanir [17] 2012	A 13-year-old girl	Bilateral submandibular masses pronounced with swallowing	US and CT	Bilateral absence of SMG and compensatory hypertrophy of the bilateral SLG

Ahmed et al. [18] 2009	A 62-year-old male	Left submandibular mass	CT	Bilateral absence of SMG and bilateral hypertrophied sublingual salivary tissue

Reija et al. [19] 2013	A 41-year-old woman	Intermittent bilateral submandibular swelling and xerostomia	US and MRI	Bilateral and symmetrical hypertrophy of both SLGs with bilateral absence of the SMGs

Shipchandler and Lorenz [20] 2008	A 60-year-old male	After resection of tumor of tongue fullness in the right submandibular region	CT	Aplasia of the left SMG with contralateral gland hypertrophy

Yilmaz et al. [21] 2010	A 41-year-old woman	Dry lips and mouth, difficulty with swallowing solid foods, heartburn, and changes in taste, over the previous 5 years	US, CT, and MRI	Absence of the right submandibular gland with compensatory hypertrophy of the ipsilateral SLG

CT: computerized tomography, MRI: magnetic resonance imaging, SMG: submandibular gland, SLG: sublingual gland, and US: ultrasound.
